# Relationship Between Depressive State and Treatment Characteristics of Acute Cervical Spinal Cord Injury in Japan

**DOI:** 10.2188/jea.JE20140233

**Published:** 2016-01-05

**Authors:** Yasufumi Matsuda, Tatsuhiko Kubo, Yoshihisa Fujino, Shinya Matsuda, Futoshi Wada, Atsuko Sugita

**Affiliations:** 1 Department of Preventive Medicine and Community Health, School of Medicine, University of Occupational and Environmental Health, Kitakyushu, Fukuoka, Japan; 2 Medical Informatics Division, University of Occupational and Environmental Health, Kitakyushu, Fukuoka, Japan; 3 Department of Rehabilitation Medicine, School of Medicine, University of Occupational and Environmental Health, Kitakyushu, Fukuoka, Japan; 4 Department of Psychiatry, School of Medicine, University of Occupational and Environmental Health, Kitakyushu, Fukuoka, Japan

**Keywords:** depression, spinal cord injury, administrative database

## Abstract

**Background:**

Few studies have assessed whether treatment of acute cervical spinal cord injury (SCI) patients contributes to depression.

**Methods:**

Using an administrative database, we assessed patients for whom the diagnosis was unspecified injuries of cervical spinal cord (International Classification of Diseases and Injuries-10th (ICD-10) code; S14.1). We categorized patients with codes for depressive episode (ICD-10 code; F32) or recurrent depressive disorder (F33), or those prescribed antidepressants (tricyclic, tetracyclic, Selective Serotonin Reuptake Inhibitors, Serotonin Noradrenaline Reuptake Inhibitors, Trazodone, Sulpiride, or Mirtazapine) as having a depressive state. We compared the rate of each acute treatment between the depressive state group and the non-depressive state group using chi-square tests, and a multiple logistic regression model was used to identify the association between the acute treatment and depressive state.

**Results:**

There were 151 patients who were judged to be in a depressive state, and the other 2115 patients were categorized into the non-depressive state group. Intervention of intravenous anesthesia, tracheostomy, artificial respiration, and gastrostomy had a significant positive correlation with depressive state. Multiple logistic regression analysis showed that tracheostomy (odds ratio [OR] 2.18; 95% confidence interval [CI], 1.09–4.38) and artificial respiration (OR 2.28; 95% CI, 1.32–3.93) were significantly associated with depressive state, and men had a 36% reduction in the risk of depressive state compared with women (OR 0.64; 95% CI, 0.44–0.94), whereas age, wound-treatment, all of the orthopedic procedures, intravenous anesthesia, and gastrostomy were not associated with depressive state.

**Conclusions:**

These findings suggest that tracheostomy, artificial respiration and female gender in the acute phase after cervical SCI might be associated with the development of depression.

## INTRODUCTION

Depressive symptoms in patients with spinal cord injury (SCI) are linked to negative outcomes, including pressure ulcers,^1^ urinary tract infections,^2^ and increased medical expenses.^2^^–^^5^ Probable major depression is one of the predictive factors of early mortality in people with SCI.^6^ Depressive behaviors have been shown to adversely affect short and long-term rehabilitation, including longer lengths of stay and less independence following discharge.^2^ Symptoms indicating depression are also associated with a low rate of return to work.^7^ Without aggressive treatment, those who are depressed during inpatient rehabilitation are likely to remain depressed up to 2 years after injury,^8^ and those who are despondent have an increased risk of suicide.^9^

Active treatment of depression leads to positive attitudes of patients for rehabilitation and good social integration. The effects of antidepressant on pain relief have been reported in patients with spinal cord or cerebrovascular lesions after 8 weeks.^10^

In order to prevent the onset of depression, it is important to understand who may be vulnerable to depression following SCI. Risk factors are specific for individuals and include middle age at injury (range, 25–49 years),^5^^,^^11^^,^^12^ female gender,^5^^,^^12^ those with a high school education or less,^12^ substance abuse,^3^^,^^13^ those who were married or separated,^5^^,^^11^ pre-injury history of depression,^3^ poorer subjective health,^11^^,^^13^ and lower satisfaction with life.^11^ Some risk factors for depression, including fewer days out of the house,^5^ less access to readily available transportation,^5^ more difficulty in daily role functioning,^11^^,^^12^ perceived inadequate social support, receiving more paid personal care assistance,^5^ increased expenses for general medication,^5^ and spending more hours in bed,^5^ may be influenced by the characteristics of patients with SCI but could also result from impairments after injury. Injury factors include cause of injury, severity of SCI,^12^^,^^14^ permanent neurological deficit, traumatic brain injury at initial hospitalization,^3^ and development of pain.^13^ However, during acute care and inpatient rehabilitation, few of these variables have been reported to be risk factors for depression, and most have been investigated for 1 year or more after injury for outpatients.

A cervical SCI is catastrophic damage and requires acute treatment, such as wound care, orthopedic procedures (ie, reduction, traction, and fixation), use of anesthetic (ie, intravenous anesthesia), respiratory care (ie, tracheostomy and artificial respiration), nutritional management (ie, gastrostomy), and urinary care (ie, cystostomy). Injury severity has not been found to be a risk factor for persistent pain, while preinjury anxiety and depression have been found to be risk factors.^15^ During traction, lower mood state and increased pain intensity could be caused by increased stretching of nerve roots and reduced mobility of patients.^16^ External spine fixation, such as halo-vest immobilization, has been reported to affect complications, including depressive mood.^17^ Using intravenous anesthesia, such as propofol, in the acute phase after motor vehicle accidents might be associated with the later symptoms of posttraumatic stress disorder.^18^ After tracheostomy, declining trends in patients’ mental health have been observed in a general population.^19^ Gastrostomy has been reported to be indicated in patients who have prolonged refusal to swallow due to severe depression^20^ and who require a tracheostomy and artificial respiration.^21^

Few reports have examined the characteristics associated with depression in patients with cervical SCI, especially in the acute treatment phase, or whether treatment contributes to depression in the general population or in cervical SCI patients. The aim of this cross-sectional study is to investigate whether acute treatment interventions for cervical SCI patients are associated with depression and to obtain useful information for preventing the onset of depression in SCI patients.

## METHODS

### Data source

We used the Diagnosis Procedure Combination (DPC) database. The DPC was constructed by the Japan Ministry of Health, Labour and Welfare in 2002 to contribute to the per-diem payment system using a patient case-mix classification and medical profiling. All 82 university hospitals were obliged to adopt this system, but adoption by community hospitals was voluntary. As of 2010, the annual number of cases in the database is approximately 3 million, which represents approximately 40% of all acute-care inpatient hospitalizations.^22^

The database contains administrative claims data and unique identifiers of hospitals, patient age and sex, the main diagnosis, the diagnosis for admission, the diagnosis for most resources in procedures, pre-existing co-morbidities at admission, and complications after admission, which are coded with International Classification of Diseases and Injuries-10th revision (ICD-10). In addition, this administrative database also includes each patient’s discharge status (dead or alive) and detailed medical information, such as surgical procedures, medications, and devices.

The research protocol of the study has been approved by the ethics committee of medical care and research of the University of Occupational and Environmental Health, Kitakyushu, Japan. Given the anonymous nature of the data collection process, informed consent was not required.

### Patient characteristics

We enrolled patients for whom the diagnosis was unspecified injuries of cervical spinal cord (ICD-10 code; S14.1). Enrolled patients had been discharged from the participating hospitals between April 1 and December 31, 2010. In the database, age is classified into four categories: 0–19 years, 20–39 years, 40–59 years, and 60 years and older. Male gender is reported as frequencies and percentages. We also describe frequencies and percentages of each acute treatment, including wound treatment, orthopedic procedures (closed reduction for simple fractures, closed reduction for fractures involved dislocations, halo traction, external spine fixation), use of anesthetic (intravenous anesthesia), respiratory care (tracheostomy and artificial respiration), nutritional management (gastrostomy), and urinary care (cystostomy).

### Diagnostic criteria for depressive state

We categorized the patients into depressive state and non-depressive state groups. Patients for whom complications have been encoded into depressive episode (ICD-10 code; F32) or recurrent depressive disorder (F33), and patients who were prescribed antidepressants (tricyclics, including amitriptyline, imipramine, nortriptyline, clomipramine, and amoxapine; tetracyclics, including maprotiline and mianserin; selective serotonin reuptake inhibitors [SSRIs], including paroxetine, fluvoxamine, and sertraline; serotonin noradrenaline reuptake inhibitors [SNRIs], including milnacipran and duloxetine; or others, including trazodone, sulpiride, and mirtazapine) were categorized in the depressive state group.

### Statistical analyses

The proportion of patients receiving each acute treatment was compared between the depressive state group and the non-depressive state group using chi-square tests. In additional analysis, we used multiple logistic regression models to identify the associations between acute treatments and depressive state and address potential confounding variables, such as age and sex. We first used an age- and sex-adjusted logistic regression analysis, followed by a multiple logistic regression analysis model containing the variables of age, sex, and all interventions provided for the patients in the depressive state group. A *P*-value of less than 0.05 was considered statistically significant. All statistical analyses are performed using STATA version 11.0 (Stata Corporation, College Station, TX, USA).

## RESULTS

### Patient data

Overall, 2266 individuals who sustained a cervical SCI were discharged or died after or during their initial treatment and inpatient rehabilitation between April 1 and December 31, 2010. The mean (standard deviation [SD]) age at discharge was 62.4 (17.8) years, and the median was 66 (inter-quartile range, 53–75) years (Table 1). Of all the individuals, 1713 (75.6%) were male. Wound treatment was provided to 327 (14.4%) patients. Concerning orthopedic procedures, 2 (0.1%), 4 (0.2%), 27 (1.2%), and 49 (2.2%) patients were provided with a closed reduction for simple fractures, a closed reduction for fractures involved dislocations, a halo traction, and an external spine fixation, respectively. Intravenous anesthesia was provided for 423 (18.7%) of the patients, and 86 (3.8%) and 204 (9.0%) individuals were provided with a tracheostomy and artificial respiration, respectively. Gastrostomy and cystostomy were provided for 38 (1.7%) and 8 (0.4%) patients.

**Table 1.** Demographic and acute treatment characteristics of cervical SCI patients

	Total *n* = 2266	Depressive state *n* = 151	Non-depressive state *n* = 2115	*P* value
Age, years				
0–19	78 (3.4)	1 (0.7)	77 (3.6)	0.121
20–39	196 (8.7)	13 (8.6)	183 (8.7)	
40–59	508 (22.4)	28 (18.5)	480 (22.7)	
≥60	1484 (65.5)	109 (72.2)	1375 (65.0)	
Males	1713 (75.6)	106 (70.2)	1607 (76.0)	0.110
				
Wound treatment	327 (14.4)	27 (17.8)	300 (14.2)	0.212
**Orthopedic procedures**				
Closed reduction for simple fractures	2 (0.1)	0 (0)	2 (0.1)	0.705
Closed reduction for fractures involved dislocations	4 (0.2)	0 (0)	4 (0.2)	0.593
Halo traction	27 (1.2)	4 (2.7)	23 (1.1)	0.088
External spine fixation	49 (2.2)	4 (2.7)	45 (2.1)	0.670
**Use of anesthetic**				
Intravenous anesthesia	423 (18.7)	40 (26.5)	383 (18.1)	0.010
**Respiratory care**				
Tracheostomy	86 (3.8)	20 (13.3)	66 (3.1)	<0.001
Artificial respiration	204 (9.0)	35 (23.2)	169 (8.0)	<0.001
**Nutritional management**				
Gastrostomy	38 (1.7)	8 (5.3)	30 (1.4)	<0.001
**Urinary care**				
Cystostomy	8 (0.4)	0 (0)	8 (0.4)	0.449

SCI, spinal cord injury.

Figures in parentheses indicate percentages.

### Depressive state

There were 151 patients with cervical SCI who were judged to be depressed using our criteria, and the other 2115 patients were categorized into the non-depressive state group. Of the 151 depressed patients, 25 (16.6%) were coded for a depressive episode (F32) and none were coded for recurrent depressive disorder (F33); 147 (97.4%) were prescribed an antidepressant.

### Statistical analyses

There were no significant differences between the depressive state group and the non-depressive state group in the proportion of patients receiving wound treatment or any orthopedic procedure (Table 1). The proportion of patients receiving intravenous anesthesia was significantly higher in the depressive state group than the non-depressive state group (26.5% vs 18.1%; *P* = 0.01). There were significantly higher proportions of patients who were provided tracheostomy (13.3%) and artificial respiration (23.2%) in the depressive state group than in the non-depressive state group (3.1% and 8.0%, respectively; *P* < 0.001 both). The proportion of patients receiving gastrostomy was significantly higher in the depressive state group than the non-depressive state group (5.3% vs 1.4%; *P* < 0.001). There was no significant difference in the percentage of patients receiving cystostomy between the two groups. Concerning demographic characteristics, there was no significant difference between the two groups in the proportion of patients in each age group and the proportion of male patients.

Table 2 shows the multiple logistic regression analysis of characteristics associated with depressive state. The age- and sex-adjusted logistic regression analysis revealed that patients receiving intravenous anesthesia (odds ratio [OR] 1.64; 95% confidence interval [CI], 1.12–2.40), tracheostomy (OR 4.93; 95% CI, 2.88–8.43), artificial respiration (OR 3.60; 95% CI, 2.37–5.46), and gastrostomy (OR 3.85; 95% CI, 1.71–8.70) had a significantly increased risk of being in depressive state. The multiple logistic regression analysis yielded a model containing the variables of age, sex, wound treatment, halo traction, external spine fixation, intravenous anesthesia, tracheostomy, artificial respiration, and gastrostomy. This analysis showed that tracheostomy (OR 2.18; 95% CI, 1.09–4.38) and artificial respiration (OR 2.28; 95% CI, 1.32–3.93) were associated with a significantly increased risk of being in a depressive state. Male sex was associated with a 36% reduction in the risk of being in a depressive state compared with female sex (OR 0.64; 95% CI, 0.44–0.94), whereas age and receipt of wound treatment, any orthopedic procedure, intravenous anesthesia, and gastrostomy were not associated with depressive state.

**Table 2.** Multiple logistic regression analysis of characteristics associated with depressive state

Independent variables	Age- and sex-adjusted analysis			Multivariable-adjusted analysis		
	
	Odds ratio	95% CI	*P* value	Odds ratio	95% CI	*P* value
Age				1.00	0.99, 1.01	0.450
Male				0.64	0.44, 0.94	0.023
Wound treatment	1.30	0.84, 2.00	0.24	1.20	0.76, 1.87	0.432
Halo traction	2.48	0.85, 7.30	0.10	1.66	0.54, 5.15	0.379
External spine fixation	1.31	0.46, 3.71	0.61	0.63	0.21, 1.88	0.407
Intravenous anesthesia	1.64	1.12, 2.40	0.01	1.36	0.91, 2.03	0.139
Tracheostomy	4.93	2.88, 8.43	<0.001	2.18	1.09, 4.38	0.028
Artificial respiration	3.60	2.37, 5.46	<0.001	2.28	1.32, 3.93	0.003
Gastrostomy	3.85	1.71, 8.70	0.001	1.96	0.80, 4.81	0.140

CI, confidence interval.

## DISCUSSION

Intravenous anesthesia, tracheostomy, artificial respiration, and gastrostomy for patients with cervical SCI in the acute treatment phase had a significant positive correlation with depressive state. The age- and sex-adjusted logistic regression analysis revealed that patients who received intravenous anesthesia, tracheostomy, artificial respiration, and gastrostomy also had significantly increased risks of depressive state. The multiple logistic regression model containing the variables of age, sex, and all interventions provided for the patients in the depressive state group showed significant positive correlations between tracheostomy and artificial respiration and depressive state.

The major strength of this study is the large sample size of 2266 patients with cervical SCI. The relationship between depression and interventions for SCI patients in the acute treatment phase has not been previously addressed. Based on the results of the current study, patients receiving either tracheostomy or artificial respiration may be more likely to suffer from depressive states than patients who do not receive these interventions. However, the existence of multicollinearity between tracheostomy and artificial respiration should be considered when interpreting the results; 17 (48.6%) of 35 patients in the depressive state group who were provided with artificial respiration also received tracheostomy. In the non-depressive state group, 54 (32.0%) of 169 patients who were provided with artificial respiration also received tracheostomy. It is possible that the depressive state group included patients with more severe impairment than the non-depressive state group, which would have created a bias toward overestimating the risk factors of the depressive state group. Although we addressed potential confounding variables, such as age and sex, using a multiple logistic regression model, unmeasured confounders might have caused selection bias.

Patients receiving respiratory care should be offered focused assessment and psychological care. Our findings suggest that medical providers should explain the potential complications of tracheostomy and artificial respiration to the patients and their families and frequently observe patients to prevent depression-related symptoms. Furthermore, adequate economic evaluation for psychological care, such as liaison activities and increasing the number of psychiatrists in acute-care hospitals, should be implemented to provide appropriate supportive care immediately after injury.

The current diagnostic criteria for Major Depressive Disorder (MDD) represent a clinical and historical consensus about the most important symptoms and signs of depressive illness.^23^ While substantial time and effort is spent diagnosing MDD via structured interviews, screening tests can quickly determine whether somatic symptoms are related to MDD.^24^ Most depression screening instruments, such as the Beck Depression Inventory and the Patient Health Questionnaire-9, require less than 10 minutes for able-bodied patients to complete.^11^ Although perfect concordance is not always present between self-reported data and a diagnosis of MDD, many instruments have been used as screening tests for MDD.

### Study limitations

Several limitations must be considered regarding our criteria of cervical SCI. Administrative databases provide limited information, and the nature of the data sources used for this research precluded the inclusion of many variables that have been shown to be associated with depression, including cause of injury, neurological impairment, pain, and socio-economical characteristics.

We lacked precise information about complications, since the claims database only allows up to four diagnostic codes for complications. It is possible that depressive state may not have been coded if there were other conditions that contributed to the hospital stay. As the existence of depressive state is not used as a classification key for payment, it is possible that depressive state is underreported by clinicians. However, we are unable to estimate the magnitude of underestimation. In this study, physicians may have diagnosed depression as a complication based on the presence or absence of depressive episodes. Few hospitals have psychiatrists who treat acute patients with mental disorders. Therefore, physicians may have been both more or less likely to note depressive state in patients with a history of depression. The information available in the administrative database could only serve as a proxy for depressive state; nevertheless, our results suggest that the effect of acute treatment interventions for depression among cervical SCI patients warrants further examination.

It is also possible that the existence of depressive state may be overestimated by including prescriptions for antidepressants in the diagnostic criteria. Antidepressants are approved by the U.S. Food and Drug Administration for the treatment of depression, anxiety disorder, panic disorder, and other conditions.^25^ Additionally, non-approved uses of antidepressants have been documented for duodenal or peptic ulcer disease, phobic disorders, and posttraumatic stress disorder. In this study, we could not distinguish between antidepressants that were prescribed for depressive state resulting from acute treatments of SCI and regular prescriptions of antidepressants for patients who had a comorbid diagnosis of depression before injury. However, we consider that most patients were initially dispensed antidepressants after they were hospitalized for cervical SCI because hospital insurance claims were reported. While we do not have information on when a definitive depression diagnosis was made, we know the first day of prescription for an inpatient. Still, limitations of the administrative data may deflate the true association due to erroneous stratification. However, uncertainty in the reliability of the diagnosis would not affect the results of the between-group comparisons.

### Conclusions

The findings of our study suggest that tracheostomy, artificial respiration and female gender in the acute phase after cervical SCI might be associated with the development of a depressive state. We believe that these findings may help physicians make more informed decisions regarding psychological care of acute SCI patients.
